# Structural equation modelling of the complex relationship between toothache and its associated factors among Indonesian children

**DOI:** 10.1038/s41598-020-70104-z

**Published:** 2020-08-11

**Authors:** Abu Bakar, Valendriyani Ningrum, Andy Lee, Wen-Kuang Hsu, Rosa Amalia, Iwan Dewanto, Shih-Chieh Lee

**Affiliations:** 1 PhD Program of Biotechnology and Industry, Da-Yeh University, Dacun, Changhua Taiwan, Republic of China; 2 School of Dentistry, Baiturrahmah University, Kuranji, Padang, Indonesia; 3 Department of Food Science and Biotechnology, Da-Yeh University, No. 168, University Road, Dacun Township, Changhua County 51591 Taiwan, Republic of China; 4 Department of Preventive and Community Dentistry, Faculty of Dentistry, Universitas Gadjah Mada, Yogyakarta, Indonesia; 5 Department of Dental Public Health, School of Dentistry, University of Muhammadiyah Yogyakarta, Jl. Brawijaya, Geblagan, Tamantirto, Kasihan, Bantul, Daerah Istimewa Yogyakarta Indonesia

**Keywords:** Health care, Risk factors

## Abstract

The Indonesian family life survey (IFLS) is used for formulating various government policies. Our preliminary study using data from the IFLS showed increase in the prevalence of toothache from 2007 to 2014. Hence, a need to analyse the factors associated with toothache using structural equation modelling (SEM) for identifying the direct and indirect association of factors with toothache was evident. The objective of this study is to analyse the complex relationships between toothache and its associated risk factors. This cross-sectional study was conducted on the data obtained from the IFLS in 2014. The IFLS data pertaining to toothache and its prevalence were analysed using the STATA software, and the multifaceted relationship was analysed using SEM. The prevalence of toothache among Indonesian children was 15.55% (1,959 of 12,595). SEM showed the direct association between toothache and age (*p* < 0.001) and parent awareness of children's health conditions (*p* < 0.005) and food consumption frequency (*p* < 0.001). Parents’ education level and residential area showed an indirect association with toothache, mediated by socio-economic status and parent awareness of children's health conditions (*p* < 0.001). We identified the multifaceted relationship between toothache and the social covariates. Parents’ awareness of their children’s health conditions mediated several indirect associations, highlighting its importance.

## Introduction

Toothache could be the clinical consequence of several odontogenic causes including severe dental caries as well as of non-odontogenic factors^1–4^. The pulp, a tissue of high neural density primarily rich in sensory nerve terminals, plays the key role in mediating odontogenic toothache, responsive to external stimuli, and in detecting potential damage to the tooth^5^. A search across the MEDLINE electronic database for epidemiological studies related to dental pain (toothache) caused by dental caries (tooth decay) showed that toothache is highly prevalent among children, even in populations with historically low levels of dental caries. Toothache is consistently associated with dental caries experience across populations^6^. The presence of toothache affects mastication^7^, speech^8^, and concentration in studies^8,9^.

The 2007 national survey of children’s health analysed parent reported-toothaches in children and concluded that 10.7% children of the United States experienced toothache in previous 6 months^10^. In Brazil, the prevalence of toothache in children between the ages of 6 and 12 years was 39% and during the previous month was 11%^11^. A cross-sectional study set in 1,862 pharmacies in London between November 2016 and January 2017 reported that 6,915 parents visited the pharmacies seeking pain medications for their children. Most parents (65%) required the pain medications to relieve the children’s oral pain, and majority of them (40.6%) complained of toothaches^12^. Compared to these countries, Indonesia has identical characteristics of population number and human development index with Brazil. Indonesia is a developing country located in Southeast Asia with a population of more than 270 million. Our preliminary study using IFLS showed significant increase in the prevalence of toothache among Indonesian children from 2007 to 2014.

Toothache is most apparent in individuals of low socio-economic groups with limited access to dental care^6^. Older children show increased probability of presenting with toothaches^13^. Moreover, various risk factors such as level of education, smoking and alcohol habits^14^, and race/ethnicity^15^ have been associated with toothaches. Notwithstanding, the association between toothache and sex has not been identified^15^. Hence, further studies to determine the factors affecting the prevalence of toothache are required.

Several factors have been associated with toothache by bivariate analysis and multivariate regression in previous studies; however, they could not determine whether the association between toothache and these factors was direct or indirect. Further, toothache caused by untreated dental caries is concurrently affected by several variables. A theoretical framework of conventional epidemiological research analysing the social factors of health can be used in oral epidemiology. The framework proposes multifaceted causal pathways between social status and health by interconnecting material, psychosocial, and behavioural pathways. Methodological consequences such as the use of multilevel modelling, path analysis, and structural equation modelling (SEM) are suggested to explain the framework. As an update of our study, we analysed the risk factors associated with toothache using SEM. It is an analytical tool commonly used for unscrambling the complex relationships between the outcomes and their covariates^16^.

**Figure 1:**
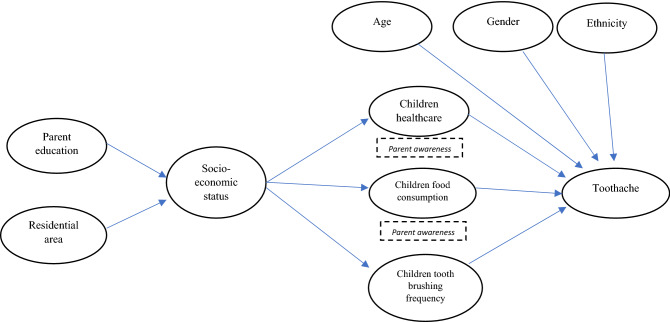
Structural equation model hypothesis of toothache and its covariates.

To explore the factors associated with toothache, we proposed an alternative model of the multifaceted relationship of parent education, residential area, socio-economic status (SES), parent awareness regarding children’s health conditions and food consumption frequency, and tooth brushing behaviour/frequency with the outcome (toothache) based on previous theoretical frameworks for dental caries^17–19^. Thus, we determined the direct and indirect relationships between toothache and its associated factors. We assumed that parent awareness regarding children's health conditions and food consumption frequency^17^ and tooth brushing behaviour/frequency^20^ were the predictors of toothache. These factors were affected by SES^17,20^. The proposed hypothesis is presented in Fig. 1.

## Results

### Participant characteristics

Records of 15,739 children in the database were eligible, of which 12,595 records were included in the study, comprising the records of 6,454 male and 6,141 female children. Of the 12,595 children, 1,959 experienced toothaches, showing a prevalence rate of 15.5%. Our preliminary study of toothache among Indonesian children showed a decrease in prevalence from 2000 to 2007; however, the prevalence showed a significant increase from 2007 to 2014 (Table 1). The summarised socio-demographic characteristics of the children and their parents and the distribution of toothaches among Indonesian children in 2014 are shown in Table 2.

**Table 1:** Preliminary study of toothache among Indonesia Children from 2000–2014.

Year	Total data*	Total used**	Toothache***	Percentage****
2000	11,686	9,268	1,145	12.35
2007	13,511	11,189	1,193	10.66
2014	15,739	12,595	1,959	15.55

*All records of toothache observations.

**Total records that could be matched to household.

***Number of children with toothaches (within the last month of the survey).

****Percentage/prevalence of children with toothaches.

**Table 2:** Summary statistics for outcome and control variables by toothache among children in Indonesia, 2014.

Outcome variables	Toothache, N = 12,595 Prevalence (%) = 15.5		
	N	Prevalence (%)	95% CI
**Age (year)**			
2–6	5,225	14.60	13.66–15.59
7–15	7,370	16.23	15.39–17.09
**Gender**			
Male	6,454	15.03	14.17–15.92
Female	6,141	16.10	15.19–17.05
**Residence area**			
Urban	7,283	15.53	14.70–16.38
Rural	5,312	15.59	14.62–16.59
**Economic status**			
Poorest—quintile 1	1,986	16.97	15.34–18.69
Quintile 2	2,385	15.97	14.53–17.51
Quintile 3	2,607	16.99	15.57–18.49
Quintile 4	2,773	15.33	14.00–16.72
Richest—quintile 5	2,844	13.12	11.90–14.41
**Parent education level**			
No education	69	18.84	10.43–30.06
Primary education	1,840	16.68	15.01–18.47
Secondary education	7,961	15.71	14.92–16.53
College	2,725	14.24	12.95–15.61
**Parent awareness on children food consumption**			
Less than once per day	44	13.64	5.17–27.35
1 to 2 times per day	3,710	16.90	15.71–18.15
3 or more times per day	8,841	15.00	14.26–15.76
**Parent awareness on children health care**			
Less than adequate	1,323	21.77	19.57–24.09
Just adequate	7,229	15.30	14.48–16.15
More than adequate	4,043	13.97	12.92–15.08
**Toothbrushing behaviour**			
In the morning	11,093	15.73	15.06–16.42
At noon	7,855	15.18	14.39–15.99
At night	3,013	17.23	15.89–18.62
After meals	380	16.84	13.22–20.99
Never	490	10.00	7.49–13.00
Don’t know	639	18.31	14.83–20.55

### Toothache distributions

As shown in Table 2, the distribution of toothache according to age, sex, residential area, SES, parent awareness of children's health conditions and food consumption frequency, and tooth brushing behaviour/frequency among Indonesian children was recorded in 2014. Toothache consistently showed higher prevalence in older children (7–15 years; 16.23%, confidence interval (CI): 15.39–17.09) compared to that in younger children (2–6 years; 14.6%, CI: 13.66–15.59). Female children showed higher prevalence of toothache (16.10%, CI: 15.19–17.05) than male children (15.03%, CI: 14.17–15.92). The prevalence of toothache was slightly higher in the rural area (15.59%, CI: 14.62–16.59) than that in the urban area (15.39%, CI: 14.70–16.38) It was also higher in children of the lower socio-economic groups (16.97%, CI: 15.34–18.69).

Predictive variables with direct or indirect correlation to toothache showed varied distributions. Table 2 demonstrates that children of parents showing higher awareness of children's health conditions showed lower tendency for experiencing toothache. However, inconclusive results for the effects of parent awareness regarding children’s food consumption frequency were reported because of the limited number of the respondents who answered that food consumption by their children was less than once per day (n: 44, CI: 5.17–27.35). The number of children who ate food 1–2 times daily was higher prevalence of toothache compared to that of children who ate less than once or three or more times daily. The survey showed that children of parents with lower education level showed a greater tendency for toothache. Children had different times of tooth brushing, and children of parents who did not know the tooth brushing behaviour of their children presented a higher prevalence of toothache. According to the data presented in Table 1, the number of children who did not brush their teeth daily was only 490 (3.8%). The number of parents who were not aware of their children's tooth brushing behaviour was only 639 (5%). Hence, it can be concluded that 91.2% Indonesian parents were aware of their children's tooth brushing behaviour. However, we observed that most children brushed their teeth in the morning (11,093) and at noon (7,885), in contrast to the universally suggested times of brushing in the night (3,013) and after meals (380).

### Toothache and ethnicity

The prevalence of toothache according to ethnicity is presented in Table 3. The prevalence of toothache among Javanese children corresponds to that among Indonesian children. The Makassar, Toraja, and Bugis ethnicities, which mostly reside on the Sulawesi Island, have consistently revealed higher toothache prevalence of more than 15% (above the nation-wide prevalence).

**Table 3:** Ethnicity and toothache among children in Indonesia, 2014.

Ethnicity of children	N	Prevalence (%)	95% CI
Javanese	4,533	15.16	14.12–16.23
Sundanese	1,387	18.24	16.24–20.38
Bali	572	15.21	12.37–18.42
Batak	628	12.26	9.80–15.08
Bugis	448	15.85	12.59–19.57
Chinese	30	6.67	0.82–22.07
Maduranese	279	18.64	14.24–23.71
Sasak	585	16.07	13.18–19.30
Minang	695	13.96	11.47–16.76
Banjar	456	17.32	13.96–21.12
Bima-Dompu	295	8.81	5.84–12.65
Makassar	228	17.98	13.22–23.59
Nias	64	25.00	15.02–37.40
Palembang	145	15.17	9.76–22.07
Sumbawa	64	12.5	5.55–23.15
Toraja	81	25.93	16.82–36.86
Betawi	573	17.28	14.27–20.63
Melayu	107	8.41	3.92–15.37
Komering	20	10.00	1.23–32.70
Banten	41	12.20	4.8–26.20
Others	954	16.25	13.96–18.74

### Direct association between age, parent awareness of children’s health conditions, and food consumption frequency and toothache

**Figure 2:**
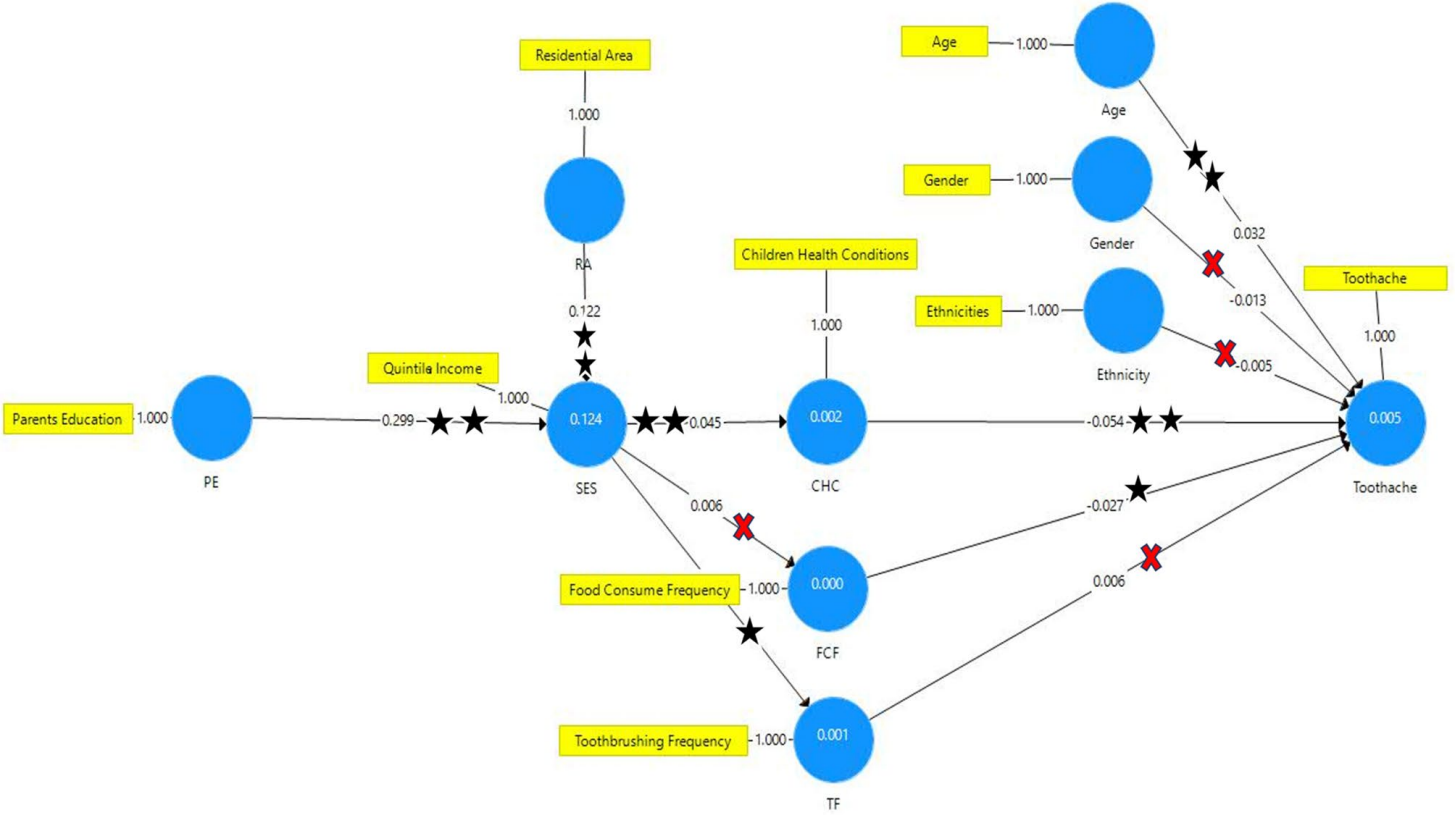
Structural equation model analysis,  : the link is supported (*p* < 0.005),  the link is supported (*p* < 0.001),  : The link is not supported.

Figure 2 shows that SEM is a reliable method of analysis. Chronbach’s alpha of each variable used in the analysis showed high reliability (r: 1.000). Table 3 presents the results of intercorrelation between the covariates and outcomes using SEM. Toothache experiences were common in children aged 7–15 years (original sample (O): 0.032, standard deviation (SD): 0.009, *p* < 0.001) and were directly affected by parent awareness of children’s food consumption frequency (O: − 0.027, SD: 0.009, *p* < 0.005) and health conditions (O: − 0.054, SD: 0.009, *p* < 0.001). The results demonstrated that children of parents who were more aware of their food consumption frequency and health conditions tended to show lower tendency for toothache.

### Indirect association between parent education level, residential area, and SES and toothache

Parent education level had an indirect, though significant, association with toothache (O: − 0.001, SD: 0.000, *p* < 0.001). Higher education level of parents was associated with higher SES (increasing parent income). Parents with good SES were more aware of their children's health conditions (O: 0.045, SD: 0.009, *p* < 0.001). Hence, their children showed lower tendency for toothache. An indirect association was also found between the residential area and toothache tendency because of the similar mediating mechanism (O: 0.000, SD: 0.000, *p* < 0.001). Children living in rural areas showed higher tendency for toothache.

## Discussion

Toothache has a variety of causes, of which dental caries is the most common^21^. A previous study reported that children with toothaches showed a 6.3-times higher probability of dental caries^22^. Most discussions related to the prevalence of toothache, its mechanism, and associated risk factors are linked to dental caries. Moreover, though our study analysed the prevalence and predictors of toothache, it may actually represent the caries experience among Indonesian children.

We investigated the prevalence of toothache among Indonesian children in this study. The results showed the prevalence of toothache in 2014 as 15.55%. This result is slightly higher than that of two previous studies, which reported a prevalence of 10.7% in US children and 11% in Brazilian children^10,14^. However, it is less than the results obtained by Bianco et al., who reported the prevalence of toothache as 23.5% in 11–16-year-old Italian children during the previous 3 months^7^. The increase in prevalence from 2007 to 2014 can be explained by SEM, which highlights the multifaceted nature of toothache.

SEM showed that the prevalence of toothache was significantly higher in older children compared to that in younger children. This result corroborates with that of a previous study that revealed higher probability of toothache in older children compared to that in younger children^13^. The association between toothache and age was also sought by Yuen et al., who found higher probability of toothache with the increase in children’s age^23^, and this relationship is observed until the time both primary and permanent teeth are exposed in the oral cavity.

Our investigation did not find an association between sex and toothache. This finding is in agreement with that of the study performed by Ortiz et al.^13^, but different from that of the studies performed by Boeira et al.^21^, Nomura et al.^24^, and Amalia et al.^25^. In our study, significant indirect association was observed between residential area and toothache. This association was mediated by SES and parent awareness of children's health conditions. Similar associations were reported by a previous study using SEM; place of residence, sex, and sugar consumption are associated with dental caries experience in 12-year-old children^25^.

A direct association was found between parent awareness of children’s food consumption frequency and toothache, though children’s food consumption frequency was not affected by SES. The association between children’s food consumption frequency and toothache, particularly sugar consumption, can be explained by the pathophysiology of dental caries. Dental caries is primarily dependent on the existence of fermentable sugars, favourable environments, cariogenic bacteria, and conducive host factors^26^.

Table 4 demonstrates the significant indirect correlation between parent education level and toothache. The results are in agreement with a previous study that did not find a direct association between mother’s education level and dental caries. Mother’s education level is significantly associated with sugar consumption and tooth brushing behaviour. The indirect association between mother’s education level and dental caries is mediated by sugar consumption. However, no significant relationship was observed between tooth brushing behaviour and dental caries^25^. These results are supported by those of our study that no significant relationship was evident between toothache and tooth brushing behaviour/frequency, which could be attributed to the fact that toothache is not related to tooth brushing frequency only. The tooth brushing technique also plays a creditable role in the association. The second possible reason could be the information available in the IFLS. The survey obtained data according to the time of tooth brushing such as in the morning, in the night, after meals, and never. This categorisation according to the time of tooth brushing could lead to inaccurate responses by the respondent as ‘two times’, in the morning and after a meal, when children might actually brush only once a day. Another point that should be considered is that significantly more children brush their teeth at noon and in the morning than in the night and after meals.

**Table 4:** Path coefficient relationship.

	Original sample (O)	SD	2.5%	97.5%	*p* values
**Direct relationship**					
Age -> Toothache	0.032	0.009	0.016	0.049	0.000**
Children Health Conditions -> Toothache	− 0.054	0.009	− 0.072	− 0.036	0.000**
Ethnicity -> Toothache	− 0.005	0.009	− 0.023	0.011	0.418
Food Children Frequency -> Toothache	− 0.027	0.009	− 0.047	− 0.009	0.003*
Gender -> Toothache	− 0.013	0.009	− 0.033	0.004	0.086
Parents Educations -> SES	0.208	0.008	0.197	0.218	0.000**
Residential Area -> SES	0.085	0.009	0.073	0.097	0.000**
SES -> Children Health Conditions	0.045	0.009	0.026	0.062	0.000**
SES -> Food Children Frequency	0.006	0.009	− 0.011	0.024	0.328
SES -> Toothbrushing Frequency	0.030	0.009	0.011	0.047	0.001*
Toothbrushing Frequency -> Toothache	0.006	0.010	− 0.013	0.025	0.364
**Indirect Relationships**					
Parents Education -> SES -> Children Health Conditions	0.013	0.003	0.008	0.018	0.000**
Residential Area -> SES -> Children Health Conditions	0.005	0.001	0.003	0.008	0.000**
Parents Education -> SES -> Food Consume Frequency	0.002	0.003	− 0.003	0.007	0.328
Residential Area -> SES -> Food Consume Frequency	0.001	0.001	− 0.001	0.003	0.328
Parents Education -> SES -> Toothbrushing Frequency	0.009	0.003	0.003	0.014	0.001*
Residential Area -> SES -> Toothbrushing Frequency	0.004	0.001	0.001	0.006	0.002*
Parents Education -> SES -> Children Health Conditions -> Toothache	− 0.001	0.000	− 0.001	− 0.000	0.000**
SES -> Children Health Conditions -> Toothache	− 0.002	0.001	− 0.004	− 0.001	0.000**
Residential Area -> SES -> Children Health Conditions -> Toothache	− 0.000	0.000	− 0.000	− 0.000	0.000**
Parents Education -> SES -> Food Consume Frequency -> Toothache	− 0.000	0.000	− 0.000	0.000	0.347
SES -> Food Consume Frequency -> Toothache	− 0.000	0.000	− 0.001	0.000	0.347
Residential Area -> SES -> Food Consume Frequency -> Toothache	− 0.000	0.000	− 0.000	0.000	0.347
Parents Education -> SES -> Toothbrushing Frequency -> Toothache	0.000	0.000	− 0.000	0.000	0.388
SES -> Toothbrushing Frequency -> Toothache	0.000	0.000	− 0.000	0.001	0.389
Residential Area -> SES -> Toothbrushing Frequency -> Toothache	0.000	0.000	− 0.000	0.000	0.388

*Significant association (*p* value < 0.005).

**Significant association (*p* value < 0.001).

A previous study reported higher dental caries experience and poorer oral hygiene in children of parents with lower SES^27^. Our study found a significant indirect association between toothache and SES. The correlation was mediated by parent awareness of children's health conditions. Moreover, in 2014, the poorest-lowest quintile group showed a tendency for toothache. This finding strongly suggests that parent awareness of children's health conditions and SES should be considered while determining the association of some covariates such as residential area and parent education level with toothache. Children of parents with lower SES showed a higher tendency for toothache because of the limited access to dental care^6^, and toothache is often a result of un-restored dental caries.

The prevalence of toothache varied according to ethnicity. This result can be explained by the special behaviours, consumption of specific foods, and varied health beliefs across ethnicities. Ethnicity plays a significant role in diet due to the effect of traditions. The Javanese population, mostly from Central Java, is known to consume limited portions of food, especially staple foods, fruits, and vegetables compared with other ethnic groups in the country^28^. A previous cross-sectional study of dental caries among children in South Sulawesi found higher prevalence (more than 15%) of early childhood caries in the Bugis, Toraja, and Javanese ethnicities compared to that in another ethnicities^29^. Interestingly, the Toraja ethnicity showed the highest toothache prevalence in 2014 of 25.93%.

Some significant limitations of our study include its cross-sectional design and the exclusion of dental caries assessment. A cross-sectional study is a snapshot survey that may have several weaknesses in relation to analysing the behaviour over a period. Further, it cannot determine any causal effect, because the predictors and outcome are analysed in a particular period. However, this particular issue is not of significant concern to our study. Previous studies also used cross-sectional design to analyse the causal effects of toothache. Furthermore, our study used data from a survey that analysed the outcome of toothache in 2014. Hence, the samples of our study were representative, which included 12,595 children. The absence of data pertaining to dental caries may affect the association between some risk factors and toothache. Dental decay could serve as the mediator variable between toothache and some predictors^7,30^. However, several previous investigations did not include dental caries experience in their survey^10,13,31^. Another minor limitation is the recall bias arising when parents were asked about their children's experience of toothache in the past four weeks. It is noteworthy that parents may have difficulties in recalling the time of toothache in their children. Notwithstanding, toothache in children is not easily ignore^12^, and most parents pay considerable attention to this oral disease and hence, are more likely to recall their children’s toothache experience.

In conclusion, toothache, usually caused by severe, untreated dental caries, affects daily activities. Our study analysed the prevalence of toothache among Indonesian children in 2014. The prevalence of toothache among children in Indonesia was 15.5%. Using SEM, we found an alternative model of the complex relationships between toothache and its associated risk factors. Previous studies have elaborated the use of SEM in determining the direct and indirect relationships between the outcome and its risk factors. Parent awareness of children’s health conditions mediated several indirect associations. Our findings strongly suggest that parents should be aware of the health conditions and food consumption frequency of their children. We also recommend the government to institute oral health education measures among parents and to develop a preventive and therapeutic programme to improve oral status among children living in rural areas.

## Methods

### Study design

The cross-sectional survey, conducted in 2014, was performed by the Research and Development in the US, which collaborated with the Population Research Centre, University of Gadjah Mada. The survey consisted of open-source data, which was available online. The survey collected individual, family, and community level data using multistage-stratified sampling. The subjects were children 2–15 years of age.

### Variable selection

The primary variable was data pertaining to toothache experience during the previous four weeks, which was obtained from the IFLS survey question, “Did your child experience toothache in the last four weeks?”. Additionally, potential covariates hypothesised to be associated with toothache included age, sex, residential area (rural/urban), SES (parent income), parent education level, parent awareness regarding the children’s health conditions and food consumption frequency, race/ethnicity, and tooth brushing behaviour/frequency. The survey question analysing tooth brushing behaviour was “When does [CHILD’S NAME] brush his/her teeth?”, and the choices were (A) in the morning, (B) at night, (C) in the afternoon, (D) after meals, (E) never, and (F) don’t know.

### Data analysis

A structural equation model was used to analyse the independent effects of the covariates after adjusting for the effects of confounders on the outcome of toothache (no = 0, yes = 1). We also categorised the independent variables into two groups and performed the labelling according to the proposed hypothesis. The following predictors were analysed: age (2–6 years old = 0, and 7–15 years old = 1), sex (male = 1, and female = 0), residential area (rural = 1, and urban = 0), economic status (quintiles 1 and 2 were categorised as poor = 1, and quintiles 3, 4, and 5 were categorised as rich = 0), parent education level (no education and only primary education were categorised as lower education level = 1, and secondary and college-level education were categorised as higher education = 0), parent awareness of children’s food consumption frequency (less than 3 per day = 1, and 3 or more per day = 0), parent awareness of children’s health conditions (less than adequate and don't know = 1, and just adequate and more than adequate = 0), and tooth brushing behaviour (once per day or never = 1, and twice per day or more = 0).

All obtained data were analysed using the STATA software, version 16.0. The STATA survey commands were used to conform to the multifaceted survey design. The population weights were entered in the data files to obtain the population-level estimates of the outcome and CI (95%). A descriptive analysis was performed to evaluate the distributions of the covariates and the outcome (toothache) in the previous four weeks. In addition, multivariable logistic regression analysis was directed to the outcome of the recent episode of toothache. The association between predictors, mediator variables, and outcomes from all variables was analysed using a structural equation model. We used our hypothesis model to analyse the direct and indirect relationships of the predictors and outcomes variables (Fig. 1). SEM was conducted using the Smart PLS version 3.2.8 [21]^32^ to assess *p* value (*p* < 0.005 and *p* < 0.001), SD, and CIs (2.5% and 97.5% CI).

### Ethics consideration

All procedures were accurately reviewed and approved by the institutional review boards (IRBs) in the US and Indonesia at the University of Gadjah Mada. The informed consents were obtained from parents and legally authorized representatives of all the illiterate parents. The informed consents were provided before the initiation of the fieldwork. The statements of anonymity and secrecy had been completed before of the beginning of survey. All study procedure was conducted in accordance with the principles of the Declaration of Helsinki.

## Supplementary information

Supplementary information.

## Data Availability

https://www.rand.org/well-being/social-and-behavioral-policy/data/FLS/IFLS/ifls5.html.
