# Supplementary material for: Structural equation modelling of the complex relationship between toothache and its associated factors among Indonesian children
Source: Sci Rep. 2020 Aug 11;10:13567. doi: 10.1038/s41598-020-70104-z (PMC7419561; doi:10.1038/s41598-020-70104-z)
Supplement: Supplementary file 1 — Supplementary information. [file 41598_2020_70104_MOESM1_ESM.pdf]

Structural Equation Modelling of the Complex Relationship Between Toothache and its Associated Factors Among Indonesian Children

Abu Bakar[1,2], Valendriyani Ningrum[2], Andy Lee[3], Wen-Kuang Hsu[3], Rosa Amalia[4], Iwan Dewanto[5*], Shih-Chieh Lee[3*]

[1]PhD Program of Biotechnology and Industry, Da-Yeh University, Dacun, Changhua, Taiwan, Republic of China

[2]School of Dentistry, Baiturrahmah University, Kuranji, Padang, Indonesia

[3]Department of Food Science and Biotechnology, Da-Yeh University, Dacun, Changhua, Taiwan, Republic of China

[4]Department of Preventive and Community Dentistry, Faculty of Dentistry, Universitas Gadjah Mada, Yogyakarta, Indonesia

[5]Department of Dental Public Health, School of Dentistry, University of Muhammadiyah Yogyakarta, Kasihan, Bantul, Indonesia

***Equal contribution corresponding authors**

1. Prof. Shih-Chieh Lee

Department of Food Science and Biotechnology, Da-Yeh University

No. 168, University Road, Dacun, Changhua, 51591, Taiwan, Republic of China

Telp: +886 4 8511888 ext. 4262, 2280

Fax: +886 4 8511320

Email: slee@mail.dyu.edu.tw or toronto.jacklee@gmail.com

2. Dr. Iwan Dewanto

Department of Dental Public Health, School of Dentistry, University of Muhammadiyah Yogyakarta

Jl. Brawijaya, Geblagan, Tamantirto, Kasihan, Bantul, Daerah Istimewa Yogyakarta, Indonesia

Telp: +62 811 2646 515

Fax: +62 274 387646

Email: iwanjoedewanto@gmail.com

Supplementary Information Legend

Supplementary information 1. SEM analysis of toothache and associated factors

Supplementary information 2. Final results of path coefficients

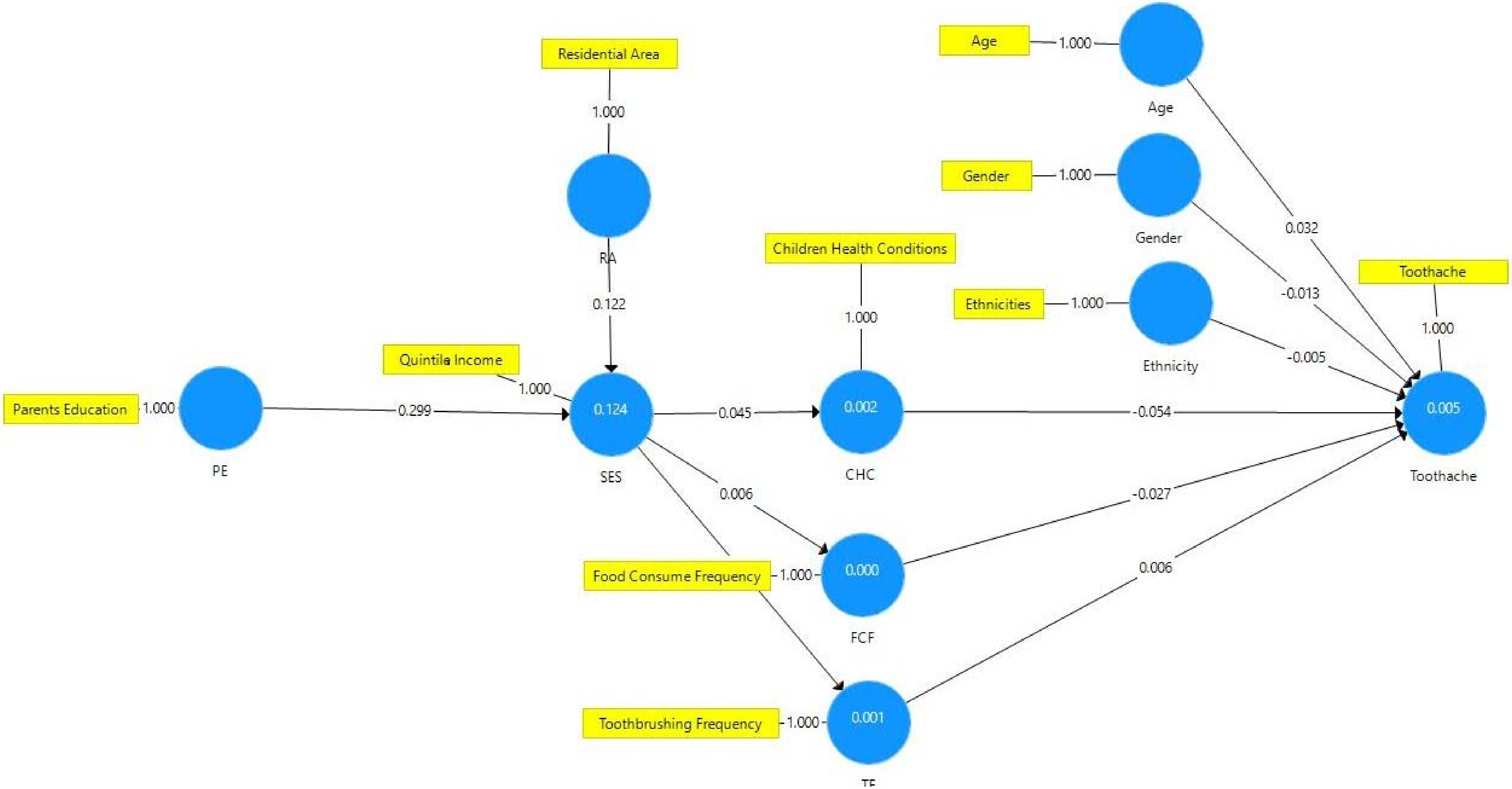

Supplementary information 1. SEM analysis of toothache and associated factors

Supplementary information 2. Final results of path coefficients

Mean, STDEV, T-Values, P-Values

	Original Sample (O)	Sample Mean (M)	Standard Deviation (STDEV)	T Statistics (|O/STDEV|)	P Values
**Age -> Toothache**	0,032	0,032	0,009	3,667	**0,000**
**CHC -> Toothache**	-0,054	-0,054	0,009	5,786	**0,000**
**Ethnicity -> Toothache**	-0,005	-0,005	0,009	0,521	**0,602**
**FCF -> Toothache**	-0,027	-0,027	0,009	2,935	**0,003**
**Gender -> Toothache**	-0,013	-0,013	0,009	1,540	**0,124**
**PE -> SES**	0,299	0,298	0,008	35,209	**0,000**
**RA -> SES**	0,122	0,121	0,009	13,838	**0,000**
**SES -> CHC**	0,045	0,045	0,009	5,042	**0,000**
**SES -> FCF**	0,006	0,006	0,009	0,719	**0,472**
**SES -> TF**	0,030	0,030	0,009	3,268	**0,001**
**TF -> Toothache**	0,006	0,007	0,010	0,637	**0,524**

Confidence Intervals

	Original Sample (O)	Sample Mean (M)	2.5%	97.5%
**Age -> Toothache**	0,032	0,032	0,016	0,049
**CHC -> Toothache**	-0,054	-0,054	-0,072	-0,036
**Ethnicity -> Toothache**	-0,005	-0,005	-0,023	0,011
**FCF -> Toothache**	-0,027	-0,027	-0,047	-0,009
**Gender -> Toothache**	-0,013	-0,013	-0,033	0,004
**PE -> SES**	0,299	0,298	0,283	0,314
**RA -> SES**	0,122	0,121	0,105	0,139
**SES -> CHC**	0,045	0,045	0,026	0,062
**SES -> FCF**	0,006	0,006	-0,011	0,024
**SES -> TF**	0,030	0,030	0,011	0,047
**TF -> Toothache**	0,006	0,007	-0,013	0,025

Confidence Intervals
Bias Corrected

	Original Sample (O)	Sample Mean (M)	Bias	2.5%	97.5%
**Age -> Toothache**	0,032	0,032	0,000	0,014	0,047
**CHC -> Toothache**	-0,054	-0,054	-0,001	-0,069	-0,035
**Ethnicity -> Toothache**	-0,005	-0,005	0,000	-0,023	0,011
**FCF -> Toothache**	-0,027	-0,027	0,000	-0,047	-0,010
**Gender -> Toothache**	-0,013	-0,013	0,000	-0,034	0,004
**PE -> SES**	0,299	0,298	-0,001	0,283	0,315
**RA -> SES**	0,122	0,121	-0,001	0,106	0,140
**SES -> CHC**	0,045	0,045	0,000	0,025	0,061
**SES -> FCF**	0,006	0,006	0,000	-0,011	0,024
**SES -> TF**	0,030	0,030	0,001	0,010	0,047
**TF -> Toothache**	0,006	0,007	0,000	-0,012	0,026
